# Phase 1 study of trebananib (AMG 386), an angiogenesis targeting angiopoietin-1/2 antagonist, in Japanese patients with advanced solid tumors

**DOI:** 10.1007/s00280-012-2000-1

**Published:** 2012-11-03

**Authors:** Toshihiko Doi, Atsushi Ohtsu, Nozomu Fuse, Takayuki Yoshino, Makoto Tahara, Kazuhiro Shibayama, Takatoshi Takubo, David M. Weinreich

**Affiliations:** 1 National Cancer Center Hospital East, 6-5-1 Kashiwanoha, Kashiwa, Chiba 277-8577 Japan; 2 Takeda Bio Development Center Ltd., Sapia Tower, 1-7-12 Marunouchi, Chiyoda-ku, Tokyo, 100-0005 Japan; 3 Amgen Inc., One Amgen Center Drive, Thousand Oaks, CA 91320-1799 USA

**Keywords:** Trebananib, AMG 386, Angiopoietin 1/2-neutralizing peptibody, Clinical trial, phase 1, Pharmacokinetics, Safety

## Abstract

**Purpose:**

To evaluate the safety, tolerability, pharmacokinetics, and antitumor activity of trebananib (AMG 386)—a first-in-class angiopoietin-1/2 antagonist peptide-Fc fusion protein—in Japanese patients, we conducted a phase 1, dose escalation study.

**Methods:**

Eligible patients were men or women, aged between 20 and 74 years, who had histologically or cytologically confirmed advanced solid tumors refractory to standard treatment. Trebananib (3, 10, and 30 mg/kg) was administered intravenously over 60 min in weekly cycles.

**Results:**

From June 2009 to April 2010, a total of 18 patients (6 for each dose cohort) were enrolled into the study. Trebananib was tolerated at all dose levels. No dose-limiting toxicities were observed. The most common adverse events were peripheral edema, constipation, fatigue, and pyrexia. Exposure to trebananib appeared to increase according to the dose administered. Serum clearance appeared to be similar across the dose range with the mean terminal-phase half-life ranging from 93.9 to 95.9 h. No neutralizing antibodies were detected. Tumor response was assessed in 18 patients. Of these, one patient with colon cancer in the 3-mg/kg cohort and one with bladder cancer in the 30-mg/kg cohort had partial responses as their best responses. These 2 patients were on treatment at the time of data cutoff (January 17, 2012).

**Conclusion:**

Trebananib was tolerated and showed acceptable safety profile in Japanese patients with advanced solid tumors. The pharmacokinetic profiles were similar to those in the previous studies in the United States. Trebananib also showed evidence of durable antitumor activity in some patients.

## Introduction

Angiogenesis is an essential process for tumor growth and metastasis [1, 2]. Unless angiogenesis occurs, tumor growth is limited because it is dependent on the continued supply of oxygen [3]. Thus, targeting angiogenesis represents one strategy for the development of anticancer therapies [4], and preclinical models of human cancer have shown that blocking angiogenesis inhibits proliferation of tumor and induces tumor regression [1, 4]. On the basis of these findings, several antiangiogenic agents have been developed and have already been approved for anticancer treatment. These agents include the inhibitors targeting vascular endothelial growth factor receptor (VEGFR) pathway [5, 6], such as monoclonal antibodies and tyrosine kinase inhibitors [7]. However, much attention has been focused on the clinical toxicity profile of these agents [8]. For example, they may increase the risk for several adverse events such as hypertension, proteinuria, coagulation disorders, and gastro-intestinal toxicity [8, 9]. Under these circumstances, newer agents are needed.

One of these candidates is an agent that blocks the interaction of angiopoietins with Tie2 receptor [10, 11]. Angiopoietin-1 (Ang1) is an angiogenic factor that signals through the endothelial cell-specific Tie2 receptor tyrosine kinase [12]. Angiopoietin-2 (Ang2) is expressed only at sites of vascular remodeling, where it reduces vascular integrity and probably makes the endothelial cells more responsive to the proliferative signals of VEGF [12]. In experimental models of cancer, imbalances between Ang1 and Ang2 resulted in a net gain of Ang2 activity, and the over-expression of Ang2 led to enhanced tumor angiogenesis and growth [13]. In addition, dual inhibition of Ang1 and Ang2 resulted in better antitumor activity than inhibition of Ang2 alone, which suggests that dual Ang1/2 inhibition is superior to selective Ang2 inhibition for suppression of angiogenesis in some postnatal settings [14]. Thus, dual Ang1/2 inhibitors are expected to be effective in the treatment of various types of cancer.

Trebananib (AMG 386) is an investigational first-in-class angiopoietin antagonist peptide-Fc fusion protein. It reduces tumor angiogenesis by selectively inhibiting the interaction of Ang1 and Ang2 with the Tie2 receptor [15]. Recently, data from 2 phase 1 studies conducted in the United States became available. In these studies, weekly administration of trebananib showed acceptable safety profile and antitumor activity as monotherapy or in combination with 3 common chemotherapy regimens in patients with advanced solid tumors [16, 17].

However, these studies mainly included Caucasians and it is uncertain whether these findings are generalizable to other ethnic populations such as Asians. Accordingly, we conducted a phase 1 study in Japan. The primary objectives of the study were to evaluate the safety, tolerability, and pharmacokinetic (PK) profile of trebananib in Japanese patients with advanced solid tumors. The secondary objectives were to explore its efficacy and potential biomarkers.

## Methods

### Study design and ethical considerations

This phase 1, open-label, dose escalation study was conducted at National Cancer Center Hospital East in Japan. The study was conducted in accordance with the Declaration of Helsinki and Good Clinical Practice. Its protocol was reviewed and approved by the institutional review board of the hospital. All patients provided written informed consent prior to their inclusion in the study.

### Patient population

Eligible patients were men or women, aged between 20 and 74 years, who had histologically or cytologically confirmed advanced solid tumor which was refractory to standard treatment or for which no curative treatment was available. Other inclusion criteria were Eastern Cooperative Oncology Group (ECOG) performance status of 0–1, normal sinus rhythm on electrocardiographic evaluation, and life expectancy of at least 3 months. Patients were also required to have adequate hematologic, renal, hepatic, and hemostatic function defined as follows: absolute neutrophil count ≥1,500/μL; platelet count ≥100,000/μL; hemoglobin ≥9 g/dL; creatinine clearance >40 mL/min; urinary protein ≤30 mg/dL in urinalysis or ≤1+ on dipstick; aspartate aminotransferase (AST) ≤2.5 times the upper limit of normal (ULN) (≤5 times ULN for patients with liver metastases); alanine aminotransferase (ALT) ≤2.5 times ULN (≤5 times ULN for those with liver metastases); alkaline phosphatase ≤2.0 times ULN (≤5 times ULN for those with bone or liver metastases); total bilirubin ≤2.0 times ULN; and prothrombin time or activated partial thromboplastin time ≤1.5 times ULN.

Patients were excluded if they had any central nervous system tumors; hematologic malignancies; unresolved toxicities from prior anticancer therapy; clinically significant cardiovascular disease within 1 year before enrollment such as myocardial infarction, unstable angina, congestive heart failure (New York Heart Association class 2–4), peripheral vascular disease, cerebrovascular disorder, transient ischemic attack, or uncontrolled arrhythmia; uncontrolled hypertension (systolic >150 mm Hg or diastolic >90 mm Hg); a history of arterial or venous thrombosis within 1 year; presence of ascites or pleural effusion requiring medical intervention; a history of bleeding diathesis or clinically significant bleeding within 6 months; non-healed wound, ulcer, or fracture; head and neck cancer; squamous cell tumor, or lung cancer with large central tumor lesions ≥3 cm; infection with human immunodeficiency virus, hepatitis C virus, or hepatitis B virus; major surgery within 4 weeks; or minor surgical procedure, placement of central venous catheter, or fine needle aspiration within 7 days. Pregnant or breastfeeding women, women of childbearing potential or men having a partner of childbearing potential who were unwilling to use adequate contraceptive precautions during the study were also excluded.

### Study treatment

Trebananib was administered intravenously over 60 (±15) min on days 1, 8, 15, and 22 without premedication. Patients were enrolled sequentially into one of 3 dose cohorts (3, 10, and 30 mg/kg; 6 patients for each cohort). The starting dose was 3 mg/kg, which was determined on the basis of the first-in-human study conducted in the United States [16]. Initially, 6 patients received trebananib intravenously every week for up to 28 days, and dose escalation proceeded unless 2 or more patients had a dose-limiting toxicity (DLT) during the first 28 days. Trebananib was not administered on day 29. For patients who had no DLTs and wished to continue the study treatment, trebananib was administered in weekly cycles after day 36.

DLT was defined as any treatment-related toxicity which met the following criteria during the first 28 days according to the National Cancer Institute Common Terminology Criteria for Adverse Events (NCI CTCAE) version 3.0: grade 4 or greater hematologic toxicity; grade 3 or greater nonhematologic toxicity other than AST, ALT, and infusion reactions; and AST or ALT >10 times ULN.

If 2 of the initial 6 patients experienced a DLT, additional 3 patients were to be enrolled at that dose level. If at least 3 of 6 patients experienced a DLT, the sponsor (Takeda Bio Development Center Ltd., Tokyo, Japan) was to discuss with the principal investigator—and with the Efficacy and Safety Evaluation Committee, if necessary—to determine whether the dose was intolerable or not.

If patients experienced any DLT during the first 28 days, treatment with trebananib was withheld and the patients were followed up until the resolution of the toxicity. If patients experienced infusion reactions, the infusion was interrupted or the infusion rate was slowed. If the infusion reaction persisted, sequential treatment with antihistamines and steroids was also allowed. Throughout the study, concomitant use of low-dose warfarin (≤1 mg/day) or low molecular weight heparin for prophylaxis of thrombosis was allowed. Other treatments were not allowed during the study except for the supportive care the investigators considered necessary.

### Assessments

Medical history was collected within 14 days before enrollment. Patients were hospitalized at least 5 days from day 1. Adverse events were monitored throughout the study and were graded according to the NCI CTCAE version 3.0. Blood pressure, pulse rate, and body temperature were measured at the following time points: predose and 1, 2, 6, 24, 48, and 96 h after starting the initial infusion at week 1; predose and 1 h after starting infusion at weeks 2–4; every week after week 6; and the end-of-study visit (i.e., 4 weeks after the end of treatment). Blood and urine samples for the laboratory tests were collected at the following time points: predose and 24, 48, and 96 h after starting the initial infusion; predose at weeks 2–4; every 4 weeks thereafter; and the end-of-study visit.

Serum samples for PK analysis were collected at the following time points: predose at weeks 1–4; 1, 2, 6, 24, 48, and 96 h after starting infusion at week 1; 1, 2, 6, 24, 48, 96, 168, and 264 h after starting infusion at week 4; every 4 weeks after week 8; and the end-of-study visit. Serum concentration of trebananib was determined by using a validated enzyme-linked immunosorbent assay [16, 17]. PK parameters were estimated by using non-compartmental methods with Phoenix WinNonlin software Version 6.1 (Pharsight Corporation, Mountain View, CA).

Serum samples for the assessment of anti-trebananib antibodies were also collected at the following time points: predose at weeks 1, 2, and 4; every 4 weeks thereafter; the end-of-study visit; and 8 weeks after the end of treatment. In the first analysis of this assessment, the presence/absence of anti-trebananib binding antibodies in serum was confirmed by using a validated acid-dissociation, bridging electrochemiluminescent immunoassay [17, 18]. Thereafter, all serum samples positive for anti-trebananib binding antibodies were evaluated for potential neutralizing capabilities in a validated in vitro receptor binding assay [17].

Furthermore, serum samples were collected for the exploration of a biomarker at predose and 48 h after starting infusion at week 1, predose at weeks 2 and 4, every 4 weeks thereafter, and the end-of-study visit. As a potential biomarker, soluble vascular cell adhesion molecule-1 (sVCAM-1) was quantified by using a specific enzyme-linked immunosorbent assay kit (Quantikine^®^; R&D Systems Inc., Minneapolis, MN) following the manufacturer’s instructions. VCAM-1 is involved in vascular remodeling, and variations in this biomarker may be indicative of a biological response to changes in the vascular endothelium [17].

Tumor response was evaluated at week 8 and every 8 weeks thereafter by the investigators using computed tomography or magnetic resonance imaging and was classified according to the Response Evaluation Criteria in Solid Tumors (RECIST) 1.0 [19].

### Statistical considerations

All data were summarized descriptively. Categorical variables are expressed as frequencies and percentages. Continuous variables are expressed as mean combined with standard deviation or median combined with range. All data were analyzed by using SAS^®^ System Version 9.1.3 (SAS Institute, Cary, NC).

## Results

From June 2009 to April 2010, a total of 18 patients (6 for each dose cohort) were enrolled into the study. All patients received trebananib and were included in the safety and efficacy analysis. Of these, one patient in the 10-mg/kg cohort discontinued the study treatment because of disease progression during the DLT evaluation period. This patient was excluded from the DLT evaluation. At the time of data cutoff (January 17, 2012), 16 patients ended the study treatment because of disease progression and 2 patients were still receiving treatment. The median number of infusions was 5.5 (range, 4–113) for 3 mg/kg, 6.0 (range, 1–17) for 10 mg/kg, and 6.0 (range, 4–92) for 30 mg/kg. The median cumulative dose was 16.50 mg/kg (range, 12.0–336.7 mg/kg), 60.00 mg/kg (range, 10.0–170.0 mg/kg), and 180.0 mg/kg (range, 120.0–2,760.0 mg/kg), respectively.

Table 1 shows the demographic and baseline characteristics of the study patients. The median age was 57.5 (range, 40–70) years in the total population. Almost all patients (94.4 %) had ECOG performance status of 0. The most common tumor types were gastric (*n* = 6; including 2 patients with gastrointestinal stromal tumors), rectal (*n* = 4; including one with rectal carcinoid), and pancreatic (*n* = 3).

**Table 1:** Demographic and baseline characteristics of the study patients

	Trebananib dose cohort			
	3 mg/kg (*n* = 6)	10 mg/kg (*n* = 6)	30 mg/kg (*n* = 6)	Total (*n* = 18)
Sex, *n* (%)				
Male	4 (66.7)	3 (50.0)	3 (50.0)	10 (55.6)
Female	2 (33.3)	3 (50.0)	3 (50.0)	8 (44.4)
Age, years				
Median (range)	57.5 (40–70)	52.5 (47–69)	63.0 (49–66)	57.5 (40–70)
Weight, kg				
Median (range)	55.90 (38.1–64.7)	65.60 (49.6–78.7)	49.65 (47.0–56.0)	55.15 (38.1–78.7)
Primary tumor type, *n* (%)				
Gastric	3 (50.0)	0 (0.0)	3 (50.0)	6 (33.3)
Rectal	1 (16.7)	2 (33.3)	1 (16.7)	4 (22.2)
Pancreatic	1 (16.7)	1 (16.7)	1 (16.7)	3 (16.7)
Colon	1 (16.7)	1 (16.7)	0 (0.0)	2 (11.1)
Bladder	0 (0.0)	0 (0.0)	1 (16.7)	1 (5.6)
Breast	0 (0.0)	1 (16.7)	0 (0.0)	1 (5.6)
Uterine	0 (0.0)	1 (16.7)	0 (0.0)	1 (5.6)
Eastern Cooperative Oncology Group performance status, *n* (%)				
0	6 (100.0)	6 (100.0)	5 (83.3)	17 (94.4)
1	0 (0.0)	0 (0.0)	1 (16.7)	1 (5.6)

Trebananib was tolerated at all dose levels. All patients had at least one adverse event, but no one discontinued the treatment because of adverse events. No DLTs were observed in any of the dose cohorts. Table 2 shows the common adverse events. The most common adverse events were peripheral edema, constipation, fatigue, and pyrexia. Grade 3 or greater adverse events were reported in 4 patients (one in the 3-mg/kg cohort, one in the 10-mg/kg cohort, and 2 in the 30-mg/kg cohort). Of these, the most frequently reported event was γ-glutamyltransferase increased (*n* = 4). No events with grade 3 or greater were considered treatment-related by the investigator.

**Table 2:** Common adverse events occurring in at least 3 patients

Preferred term	Trebananib dose cohort							
	3 mg/kg (*n* = 6)		10 mg/kg (*n* = 6)		30 mg/kg (*n* = 6)		Total (*n* = 18)	
	Any	≥Grade 3	Any	≥Grade 3	Any	≥Grade 3	Any	≥Grade 3
Edema peripheral	2 (33)	0 (0)	2 (33)	0 (0)	3 (50)	0 (0)	7 (39)	0 (0)
Constipation	2 (33)	0 (0)	1 (17)	0 (0)	2 (33)	0 (0)	5 (28)	0 (0)
Fatigue	3 (50)	0 (0)	1 (17)	0 (0)	1 (17)	0 (0)	5 (28)	0 (0)
Pyrexia	2 (33)	0 (0)	1 (17)	0 (0)	2 (33)	0 (0)	5 (28)	0 (0)
Anorexia	2 (33)	0 (0)	0 (0)	0 (0)	2 (33)	0 (0)	4 (22)	0 (0)
Diarrhea	1 (17)	0 (0)	1 (17)	0 (0)	2 (33)	0 (0)	4 (22)	0 (0)
ECOG PS worsened	3 (50)	0 (0)	0 (0)	0 (0)	1 (17)	0 (0)	4 (22)	0 (0)
γ-Glutamyl transferase increased	1 (17)	1 (17)	1 (17)	1 (17)	2 (33)	2 (33)	4 (22)	4 (22)
Hypertension	0 (0)	0 (0)	2 (33)	0 (0)	2 (33)	0 (0)	4 (22)	0 (0)
Abdominal distension	2 (33)	0 (0)	0 (0)	0 (0)	1 (17)	0 (0)	3 (17)	0 (0)
Ascites	2 (33)	0 (0)	0 (0)	0 (0)	1 (17)	0 (0)	3 (17)	0 (0)
Cancer pain	1 (17)	0 (0)	1 (17)	0 (0)	1 (17)	0 (0)	3 (17)	0 (0)
Nausea	1 (17)	0 (0)	0 (0)	0 (0)	2 (33)	0 (0)	3 (17)	0 (0)
Rash	1 (17)	0 (0)	1 (17)	0 (0)	1 (17)	0 (0)	3 (17)	0 (0)
Stomatitis	1 (17)	0 (0)	0 (0)	0 (0)	2 (33)	0 (0)	3 (17)	0 (0)

*ECOG PS* Eastern Cooperative Oncology Group performance status

Serious adverse events were reported in the following 3 patients: one in the 3-mg/kg cohort (ascites and pleural effusion), one in the 3-mg/kg cohort (subclavian vein thrombosis and cholecystitis), and one in the 30-mg/kg cohort (anorexia). Of these, cholecystitis was considered treatment-related because the patient did not have any complications, such as gallstones, which are known to be a cause of cholecystitis. Other events were not considered treatment-related by the investigator. Subclavian vein thrombosis was considered to be related to the central venous catheter that was placed in the patient.

**Fig. 1:**
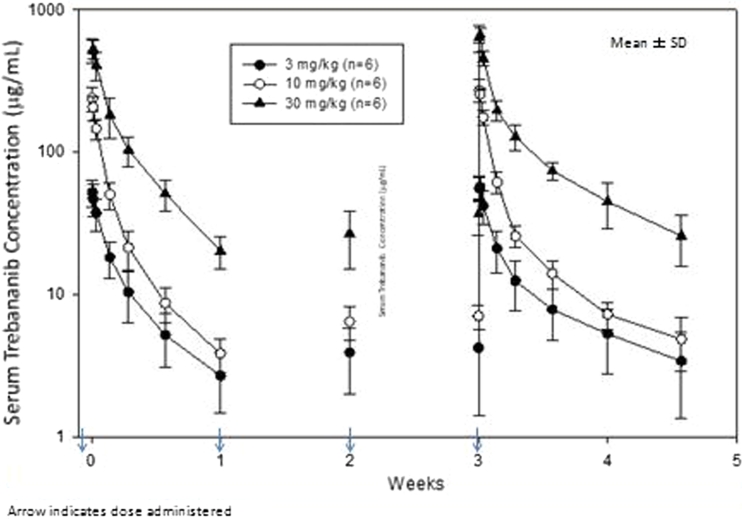
Serum concentration–time curves of trebananib

Figure 1 shows serum concentration–time profiles of trebananib. The serum concentration of trebananib gradually declined after the completion of 1-hour infusion. After 4 once-weekly infusions, the serum concentrations increased slightly compared with those after the initial infusion. Table 3 shows the PK parameters of trebananib. Exposure to trebananib (maximum observed concentration [*C*_max_] and area under the serum concentration–time curve from time 0 to 168 h post-dose [AUC_0-168_]) on both weeks 1 and 4 appeared to increase according to the dose administered. Serum clearance appeared to be similar across the dose levels with the mean total clearance ranging from 1.44 to 1.71 mL/h/kg. The mean terminal-phase half-life ranged from 93.9 to 95.9 h. Minimal accumulation was observed after multiple dosing with approximate 1.2 of the accumulation ratio of AUC_0-168_.

**Table 3:** Pharmacokinetic parameters of trebananib

Week 1			Week 4							
*T*_max_ (h)	*C*_max_ (μg/mL)	AUC_0-168_ (h*μg/mL)	*T*_max_ (h)	*C*_max_ (μg/mL)	AUC_0-168_ (h*μg/mL)	*t*_1/2, z_ (h)	CL (mL/h/kg)	*V*_ss_ (mL/kg)	AUC_0-168_ AR	*C*_min_ (μg/mL)
3 mg/kg										
*n* = 6			*n* = 6							
1.07 (1.03–1.08)	52.3 (11.3)	1,760 (582)	1.07 (1.02–2.03)	59.0 (10.1)	2,170 (715)	95.9 (35.1)	1.50 (0.423)	158 (49.0)	1.24 (0.0514)	5.32 (2.54)

10 mg/kg										
*n* = 6			*n* = 5							
1.03 (1.02–1.05)	239 (47.1)	4,630 (925)	1.02 (1.02–2.02)	277 (48.8)	5,880 (560)	95.4 (14.8)	1.71 (0.165)	121 (22.2)	1.19 (0.0686)	7.27 (1.52)

30 mg/kg										
*n* = 6			*n* = 6							
1.17 (1.02–2.00)	551 (86.8)	18,000 (4,490)	1.51 (1.03–2.02)	689 (105)	21,200 (2,910)	93.9 (25.6)	1.44 (0.191)	137 (30.3)	1.21 (0.214)	45.1 (16.2)

All parameters are reported as mean (standard deviation) values, except for *T*_max_, which is reported as a median (range) value

AUC_0-168_ = The area under serum concentration–time curve from time 0 to 168 h post-dose, AUC_0-168_ AR = The AUC_0-168_ accumulation ratio (=[AUC_0-168_ on week 4]/[AUC_0-168_ on week 1]), CL = The apparent total clearance (=[actual dose]/[AUC_0-168_ on week 4]), *C*_max_ = The maximum observed serum concentration after dosing, *C*_min_ = The serum concentration at 168 h after dosing, *t*_1/2, z_ = The estimated terminal-phase half-life (=ln(2)/λz, where λz is the terminal rate constant estimated via linear regression of the terminal log-linear decay phase), *T*_max_ = The time at which *C*_max_ was observed, *V*_ss_ = The volume of distribution at steady state (=MRT*CL, where MRT is the mean residence time.)

Anti-trebananib binding antibodies were detected in 2 patients at 3 mg/kg and one at 10 mg/kg. However, no neutralizing antibodies were observed in their serum samples. Concentrations of sVCAM-1 transiently increased after the infusion according to the dose administered (data not shown).

**Fig. 2:**
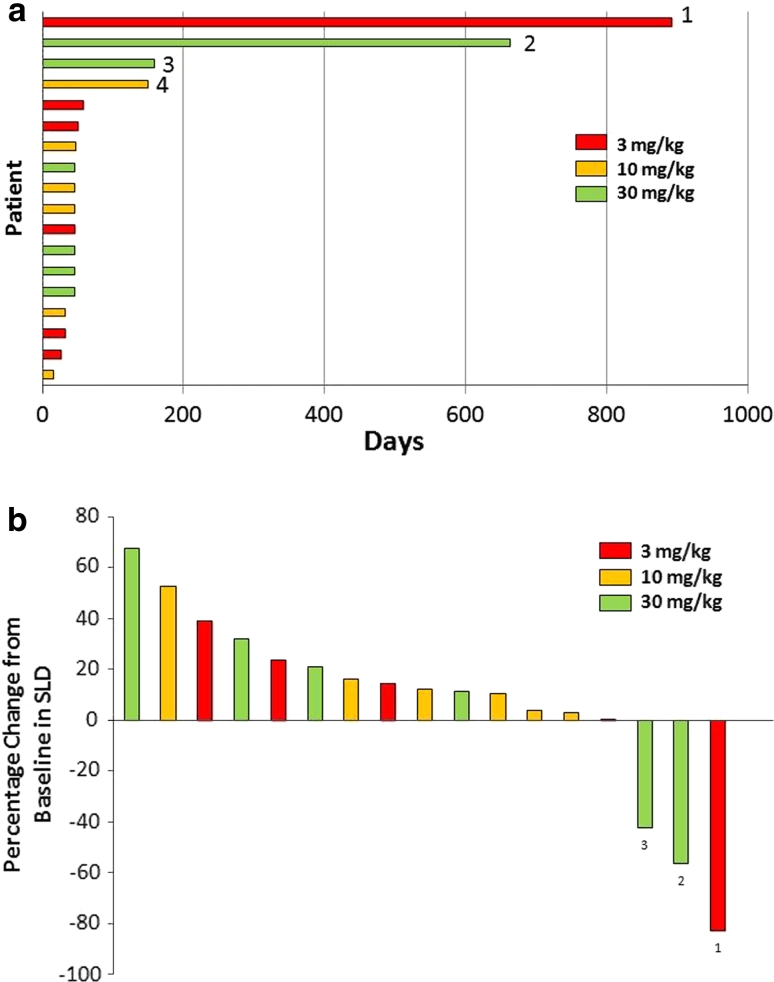
Antitumor activity of trebananib. **a** Time to disease progression. Tumor type: *1* Colon, *2* Bladder, *3* Stomach (gastrointestinal stromal tumor), *4* Pancreas. **b** The maximum percent change in target lesions. *SLD* sum of the longest diameter. Tumor type: *1* Colon, *2* Bladder, *3* Stomach (gastrointestinal stromal tumor). One patient with colon cancer in the 3-mg/kg cohort and one with bladder cancer in the 30-mg/kg cohort had a best response of partial response

Figure 2 shows the antitumor activity of trebananib. All patients had measurable diseases at baseline. One patient with colon cancer in the 3-mg/kg cohort and one with bladder cancer in the 30-mg/kg cohort had a best response of partial response. These 2 patients were on treatment at the time of data cutoff. The longest treatment period was over 2 years in the patient with colon cancer (Fig. 2a). One of 18 patients underwent computed tomography examination without receiving contrast agent at post-dose. Therefore, the tumor regions were not comparable between baseline and post-dose. As a result, 17 patients were included in the maximum percentage change in target lesions (Fig. 2b). No clinically meaningful relationship was observed between the concentrations of sVCAM-1 and tumor responses (data not shown).

## Discussion

Results of our study show that weekly infusions of trebananib up to 30 mg/kg were tolerated without any treatment discontinuation because of adverse events. Adverse events were mild to moderate in most patients. No DLTs were observed. These results are consistent with those of the phase 1 single-agent study conducted in the United States [16]. In our study, the most common toxicities included peripheral edema and fatigue, which were also observed in the study conducted in the United States [16]. Of these, peripheral edema is a unique adverse event that has been considered to be related to trebananib [20]. No unexpected toxicities were reported.

The safety profile of trebananib was different from that of the VEGF/VEGFR pathway inhibitors, although both agents inhibit angiogenesis. Of the common toxicities associated with the VEGF-axis inhibitors, hypertension is the most prominent adverse event because the VEGF/VEGFR pathway is a regulator of vasodilatation [8, 9]. For example, grade 3/4 hypertension occurred in 4–21 % of patients who received the VEGF-axis inhibitors in the previous studies [21–23]. It is also a frequent reason to delay treatment [9]. In our study, although 4 patients experienced hypertension, these events were mild to moderate and did not require treatment discontinuation. No grade 3/4 hypertension was reported. Other common toxicities associated with VEGF-axis inhibitors such as proteinuria, hemorrhage, or thrombosis did not occur. Although subclavian vein thrombosis was reported in one patient, this event was considered to be related to the central venous catheterization. These distinct safety profiles of trebananib and the VEGF-axis inhibitors are probably derived from the fact that both agents inhibit angiogenesis in a completely different pathway and suggest that they may be combined to improve efficacy without significant overlapping toxicities.

In the PK data of our study, dose-dependent exposure and minimal accumulation of trebananib after 4 once-weekly infusions were observed. These results are consistent with those of the phase 1 studies in the United States [16, 17], and estimated values of PK parameters were similar among the studies. For example, the mean serum clearance ranged from 1.44 to 1.71 mL/h/kg in our study, whereas it ranged from 0.70 to 1.27 mL/h/kg in the previous single-agent study [16]. In addition, the mean *C*_max_ after 4 once-weekly infusions of 10-mg/kg trebananib was 277 μg/mL in our study, 249 μg/mL in the single-agent study [16], and 219 μg/mL in the study combined with chemotherapies [17]. These results suggest the absence of ethnic difference in the PK profile of trebananib when intravenously administered weekly.

Although anti-trebananib binding antibodies were detected in 3 patients in our study, no neutralizing antibodies were detected. The previous studies have provided similar results and have also shown that the anti-trebananib antibodies had no apparent effect on serum trebananib concentrations [16, 17]. From these results, we consider that the immune response induced by multiple dosing of trebananib is unlikely to affect the exposure.

In the efficacy analysis, trebananib showed evidence of antitumor activity. Two patients, one with colon cancer and the other with bladder cancer, achieved a partial response. Both of them had a durable partial response and were on treatment at the time of data cutoff. In the previous single-agent study conducted in the United States, of 29 patients with evaluable tumor response, one patient with advanced ovarian cancer refractory to multiple chemotherapies had a partial response with the dose of 30 mg/kg [16]. These results suggest the efficacy of trebananib as monotherapy. Although concentrations of sVCAM-1 transiently increased in a dose-dependent manner, no clinically meaningful relationship was observed between the concentrations of sVCAM-1 and tumor responses. Further efforts may be warranted, because selecting suitable biomarkers for angiopoietin/Tie2 axis is still challenging [17].

In conclusion, trebananib was tolerated and showed acceptable safety profile in Japanese patients with advanced solid tumors. These results are consistent with those of the phase 1 single-agent study conducted in the United States. The PK parameters in Japanese were also similar to those obtained in the previous studies in the United States. These results suggest the absence of ethnic difference. Furthermore, trebananib showed evidence of durable antitumor activity in some patients. To confirm the favorable profiles of trebananib, further clinical trials including randomized controlled trials are needed. At present, several trials that evaluate the efficacy and safety of trebananib in combination with either VEGF-axis inhibitors or chemotherapies are in progress [24]. These programs include 3 phase 3 clinical trials in patients with ovarian cancer (TRINOVA-1, TRINOVA-2 and TRINOVA-3; ClinicalTrials.gov NCT01204749, NCT01281254 and NCT01493505, respectively).

## Abbreviations

VEGFR: Vascular endothelial growth factor receptor
Ang1: Angiopoietin-1
Ang2: Angiopoietin-2
PK: Pharmacokinetic
ECOG: Eastern Cooperative Oncology Group
AST: Aspartate aminotransferase
ULN: Upper limit of normal
ALT: Alanine aminotransferase
DLT: Dose-limiting toxicity
NCI CTCAE: National Cancer Institute Common Terminology Criteria for Adverse Events
sVCAM-1: Soluble vascular cell adhesion molecule-1
*C*_max_: Maximum observed concentration
AUC_0-168_: Area under the serum concentration–time curve from time 0 to 168 h post-dose
